# Healthcare Utilization Among Youth with Chronic Illness Receiving Care at a Large Urban Academic Healthcare System

**DOI:** 10.3390/healthcare12222278

**Published:** 2024-11-14

**Authors:** William Daniel Soulsby, Linda S. Franck, Emily Perito, Paul Brakeman, Addison Cuneo, Laura Quill, John Boscardin, Emily von Scheven

**Affiliations:** 1 Wellness Center for Youth with Chronic Conditions, University of California, San Francisco, CA 94143, USAevonsche@ucsf.edu (E.v.S.); 2 Department of Pediatrics, School of Medicine, University of California, San Francisco, CA 94143, USA; 3 Department of Family Health Care Nursing, School of Nursing, University of California, San Francisco, CA 94143, USA; 4 Department of Epidemiology and Biostatistics, School of Medicine, University of California, San Francisco, CA 94143, USA

**Keywords:** chronic illness, healthcare utilization, health disparities, social determinants of health

## Abstract

Background/Objective: We sought to understand healthcare utilization and barriers to care among youth with chronic illness who interact frequently with the healthcare system. Methods: This was a retrospective analysis of healthcare utilization for youth ≤25 years of age with chronic illness during one calendar year (1 January 2021–31 December 2021) in a single urban academic healthcare system. Inclusion criteria were (1) having at least one healthcare encounter in the calendar year of 2021 and (2) having at least six healthcare encounters over the preceding 3-year period or having a qualifying chronic illness. Demographic and clinical characteristics were collected along with self-reported and derived social determinants of health. Univariable and multivariable regression models were created to identify predictors of missed clinic visits, telehealth use, and activated patient portal accounts. Results: The cohort (N = 14,245) was demographically, clinically, and socioeconomically diverse. The youth had frequent clinic visits (median 9, IQR 4–18), multiple subspecialty care referrals (median 4, 1–8), were prescribed multiple medications (median 6, 3–10), and a high proportion received emergency department (18%) or inpatient treatment (15%). Race and public insurance were significant predictors of missed clinic visits and telehealth use. Primary language was a significant predictor of patient portal activation. Conclusions: Youth with chronic illness who are high users of the healthcare system face a high burden of clinic, emergency room, and hospital visits, referrals, and medications. Systematic efforts to lower the healthcare burden and improve care access should address existing racial and socioeconomic disparities affecting this patient population, who are likely to need frequent healthcare over their lifetime.

## 1. Introduction

Chronic diseases, including conditions such as diabetes and asthma, are increasing in prevalence in the pediatric population. More than 27% of U.S. children have at least 1 chronic medical condition, and 1 in 15 have multiple chronic medical conditions [1]. As advances in healthcare have improved survival for children with previously fatal conditions, these numbers continue to rise [2]. Children with chronic medical conditions require ongoing complex care, including lifelong medical management and frequent interactions with the healthcare system. This significantly impacts the child’s and their family’s quality of life and long-term health outcomes [3,4]. Healthcare utilization has been investigated for several individual chronic diseases, such as type I diabetes mellitus and inflammatory bowel disease [5,6]. However, despite their rising prevalence, the demographic and clinical characteristics and healthcare utilization patterns of children with chronic disease at large have not been well described. Understanding demographic and clinical characteristics that influence access to care can help us design more equitable and successful healthcare delivery systems for this unique population, who comprise a large proportion of pediatric care.

Of particular interest are questions about the prevalence of missed clinic visits, use of telehealth, and patient portal use, in addition to racial and other health disparities impacting these metrics among youth with chronic illness. Identification of racial disparities is of significant importance. Race is a social construct that is dynamic and defined by socially dominant groups. Therefore, racial disparities may reflect the consequences of individual and/or structural racism, rather than biological difference alone [7,8,9]. From the patient and caregiver perspective, missed visits limit opportunities to discuss persistent symptoms, evaluate medication challenges, and optimize treatments. From the health system perspective, they are associated with considerable financial loss and missed opportunities to provide care for other waitlisted patients [10]. However, predictors of missed appointments and other healthcare usage metrics for pediatric patients with chronic illness, who are high users of subspecialty practices, have not been described. With a lens towards intervention, telehealth and patient portal use offer an opportunity to remotely bridge gaps in care and limit the burden for youth with chronic illness and their families, who require frequent interaction with the healthcare system. Whether disparities affect the use of these tools among youth with chronic illness at large is unknown.

To address these knowledge gaps and as part of an institutional effort to understand healthcare utilization and barriers for children with chronic illness who interact with the healthcare system frequently, we performed an analysis to describe the demographic and socioeconomic characteristics of these youth and their healthcare utilization patterns. Additionally, we performed an exploratory analysis of factors associated with missed clinic visits, successful telehealth use, and patient portal activation within our cohort with the goal of identifying health disparities in care delivery. We hypothesized that this cohort would experience high healthcare utilization and burden with disparities among racially minoritized and socioeconomically disadvantaged youth missed clinic visits, telehealth use, and patient portal activation.

## 2. Methods

### 2.1. Design

This was a retrospective analysis of youth who are high users of healthcare at a tertiary academic medical center in a single year between 1 January 2021 and 31 December 2021. The medical center is a large academic healthcare system in the San Francisco Bay Area, a region including 9 counties and a population of over 7 million people. The medical center’s pediatric practice is anchored by two academic children’s hospitals (in San Francisco and Oakland). It is a quaternary referral center for the region. Additionally, the medical center includes 21 clinics across the Bay Area that provide pediatric and young adult outpatient care.

### 2.2. Sample

Inclusion criteria for the cohort were (1) youth ≤25 years of age who (2) had at least one healthcare encounter in the calendar year of 2021 and (3) had at least 6 healthcare encounters over the prior 3-year period (1 January 2018–31 December 2021) within the same subspecialty clinic or had a specific diagnosis associated with chronic illness (including obesity, depression, learning disability, autism, global developmental delay, attention deficit hyperactivity disorder [ADHD], eczema, asthma, trisomy 21, and anaphylaxis). The university Institutional Review Board (#21-33605) reviewed our study protocol and approved this study under expedited review. Participant consent was not required for this retrospective chart review.

### 2.3. Data Acquisition and Analysis

Demographic characteristics as reported by patients or their guardians were extracted from the electronic medical record (EMR) via proprietary software from the EMR system using Structured Query Language (SQL) code for the most recent encounter over the 1-year study period. These included age, sex assigned at birth, self-identified race (with the following categories: White, Black, Asian, American Indian or Alaska Native, Native Hawaiian or other Pacific Islander, Unknown or Declined to State, and Other, which included those who identified as more than one racial category) and ethnicity (non-Hispanic/Latine, Hispanic/Latine, or Unknown/Declined to State). Socioeconomic measures were derived from 5-year estimates from the American Community Survey (ACS) 2020 survey data from the U.S. Census Bureau [11], including median income for zip code. Urbanicity versus rurality was derived by geocoding 2010 Rural–Urban Commuting Area (RUCA) scores from 5-digit zip codes from the USDA [12]. Neighborhood social deprivation was derived by geocoding from the 5-digit zip code the Multidimensional Deprivation Index, a composite measure using U.S. Census data that encompasses 6 domains, including standard of living, health, education, economic security, housing quality, and neighborhood quality [13]. Healthcare utilization metrics included the total number of clinic visits, number of medications (at most recent encounter), number of referrals (by orders placed over the 1-year period), number of emergency department visits and inpatient hospitalizations at the medical center, patient portal (MyChart) account activation status, and any ancillary encounters (child life, nutrition, or social work).

Descriptive statistics were performed using Kruskal–Wallis and chi-squared tests, as appropriate, to describe the cohort by primary subspecialty clinic, grouped into 12 categories based on specialty relatedness and distribution of the cohort (allergy/immunology/rheumatology and infectious diseases; cardiology and pulmonology; endocrinology; hematology/oncology and bone marrow transplant [BMT]; gastroenterology and hepatology; primary care and obstetrics and gynecology; surgical subspecialties; psychiatry and mental health specialties; neurology, genetics, and physical medicine and rehabilitation; nephrology; and dermatology).

Univariate and multivariable Poisson regression models were created to investigate the association between demographic and socioeconomic factors and the total number of missed clinic visits and the total number of completed telehealth visits, as a proportion of the total number of scheduled visits. Univariate and multivariable logistic regression models were used to identify factors associated with activated patient portal (MyChart) accounts. Covariates included for adjustment in multivariable models included age, sex assigned at birth, race, Hispanic/Latine ethnicity, median income for zip code, RUCA score, primary payor, primary language, clinical site, any inpatient hospitalization, and residence in the San Francisco Bay Area. Variance inflation factor (VIF) was calculated to assess for collinearity in the models. Interaction terms were tested between race and median income and race and payor and were included in the final multivariable regression models if statistically significant (*p* < 0.05). All analyses were conducted using STATA version 16, a statistical software package used to manage and analyze data [14]. Maps were created to demonstrate the geographic distribution of subjects using ArcGIS Pro [15].

## 3. Results

### 3.1. Demographic and Clinical Characteristics

**Scheme 1:**
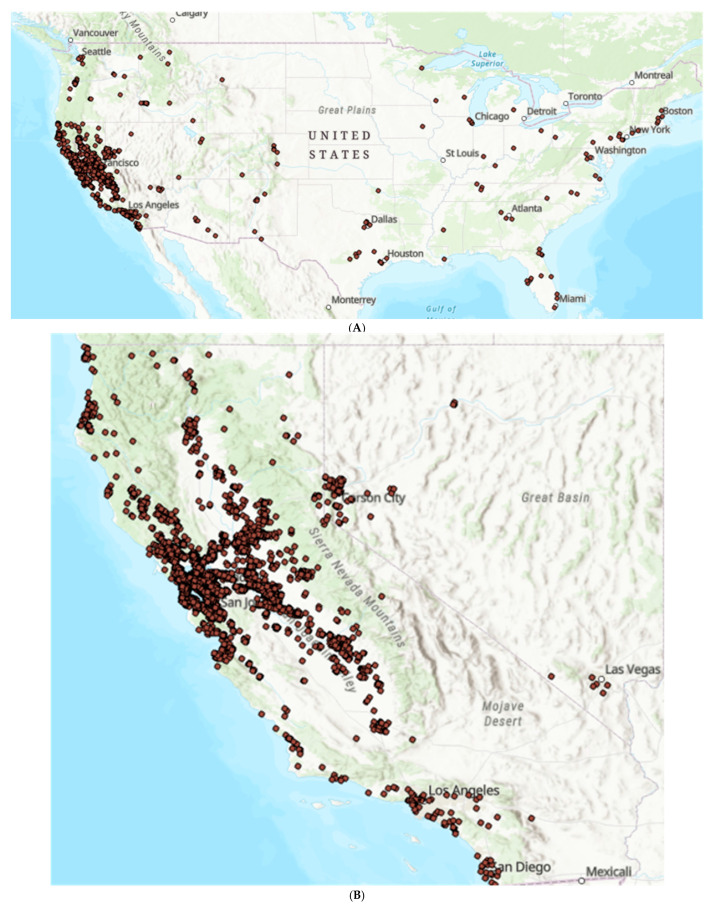
(**A**) Geographic distribution of the high healthcare use cohort of youth (up to age 25) with chronic illnesses in 2021. (**B**). Geographic distribution of the same cohort within California.

In total, 14,245 youth were identified in our cohort (Table 1), primarily residing in California (98.3%) (Scheme 1A,B). A total of 10,212 patients (71.7%) were local to the San Francisco Bay Area, a highly urban region (93% with a RUCA score between 1 and 3) with a higher median income (USD 108,680) than national averages for 2021 (USD 70,784 [16]) (Table 2).

**Figure 1:**
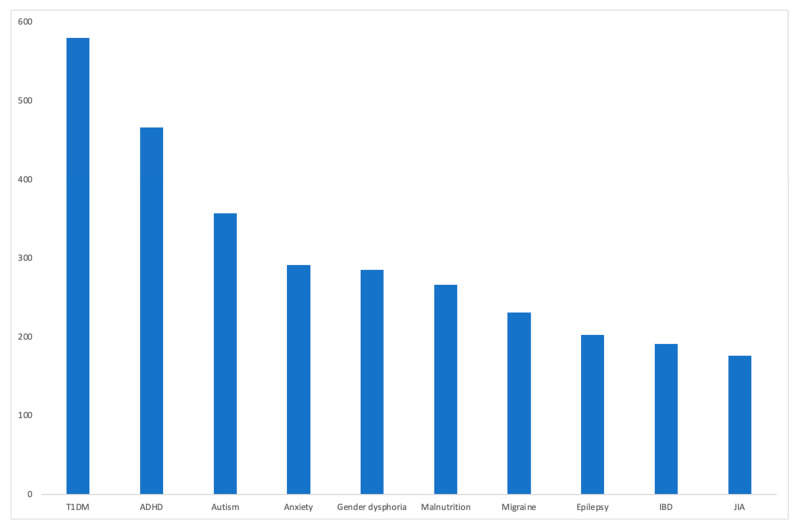
Most common diagnoses among youth (up to age 25) defined as high users of the medical system seen at University of California, San Francisco (UCSF) Health in 2021. Acronyms (T1DM = Type I Diabetes Mellitus; ADHD = Attention Deficit Hyperactivity Disorder; IBD = Inflammatory Bowel Disease; JIA = Juvenile Idiopathic Arthritis).

The most common chronic illnesses were type 1 diabetes mellitus, attention deficit hyperactivity disorder (ADHD), autism, anxiety, and gender dysphoria (Figure 1). The median age was 14 years (IQR 8–17) with 50% of individuals identifying as male (Table 1). The cohort was racially diverse with 38% identifying as White, 35% as Other (including multiracial), 12% Asian, and 10% Black. There were 33% identifying as Hispanic/Latine. Most of the cohort was English-speaking (84%), followed by Spanish-speaking (14%). A slight majority were publicly insured (52%).

### 3.2. Healthcare Utilization

The cohort comprised high users of the healthcare system (Table 3). They had multiple clinic visits within the health system (median of 9 visits/year, IQR 4–18), with some of the visits by telehealth (median of 2 visits/year, IQR 1–4; proportion 25% of all visits, IQR 4–50%). Missed clinic visits were low overall (median of 0 visits/year, IQR 0–2). Multiple specialty referrals were common (median of 4, IQR 1–8), 18% of the cohort required emergency department visits, and 15% had one or more inpatient hospitalization over the 1-year period. Overall, there was low documented use of ancillary services, such as child life, nutrition, or social work (2%, 8%, and 5%, respectively). Many medications were prescribed (median of 6, IQR 3–10). The majority of individuals had activated their patient portal accounts (69%).

### 3.3. Missed Clinic Visits

Regression modeling identified several significant demographic and socioeconomic predictors of clinic absences (Table 4). In unadjusted analysis, all racial groups were associated with more missed clinic visits as compared to White race. Also in an unadjusted analysis, Hispanic/Latine ethnicity was associated with more missed clinic visits compared to non-Hispanic/Latine ethnicity. In adjusted analysis, Black (adjusted incidence rate ratio [aIRR] 1.56, 95% CI: 1.42–1.71), Native Hawaiian or other Pacific Islander (aIRR 1.28, 95% CI: 1.01–1.62), and Other (aIRR 1.09, 95% CI: 1.01–1.18) race were associated with more missed clinic visits, as compared to White race. Hispanic/Latine ethnicity was no longer statistically significant upon adjustment for covariates. In unadjusted and adjusted analysis, California Children’s Services (CCS) and non-CCS forms of public insurance (aIRR 1.54, 95% CI: 1.42–1.68; aIRR 1.52, 95% CI: 1.40–1.65, respectively) were associated with more missed clinic visits as compared to private insurance. Additionally, lower median income for zip code was associated with more missed clinic visits, and the worst among those living in the lowest income quartile, as compared to the highest median income (aIRR 1.33, 95% CI 1.21–1.47). Interactions between race and median income for zip code and race and insurance status were not statistically significant and therefore not included in the final models. The adjusted r^2^ value for this regression model was 0.06, meaning the variables included in the model only accounted for 6% of the variability in clinic absences.

### 3.4. Telehealth Use

We also identified demographic and socioeconomic predictors of telehealth utilization (Table 5). In unadjusted analysis, individuals identifying as Asian (IRR 0.89, 95% CI: 0.82–0.95), Black (IRR 0.63, 95% CI: 0.57–0.69), Native Hawaiian or other Pacific Islander (IRR 0.73, 95% CI: 0.56–0.95), and Other (IRR 0.73, 95% CI: 0.69–0.77) race as compared to White, and Hispanic/Latine ethnicity (IRR 0.80, 95% CI: 0.75–0.84) as compared to those who were non-Hispanic/Latine had lower telehealth use. In adjusted analysis, Black as compared to White race (aIRR 0.87, 95% CI: 0.69–1.09), Hispanic/Latine as compared to non-Hispanic/Latine ethnicity (aIRR 0.96, 95% CI: 0.90–1.02), and the lowest median income for zip code quartile as compared to highest (aIRR 0.91, 95% CI: 0.81–1.02) were no longer significantly associated with reduced telehealth use. Importantly, these adjusted models included two statistically significant interaction terms between race and median income for zip code (*p* = 0.007) and race and payor (*p* = 0.04), which significantly attenuated the association between Black as compared to White race on telehealth use. In both unadjusted and adjusted analyses, public insurance from CCS was associated with statistically significantly lower telehealth use (aIRR 0.90, 95% CI: 0.83–0.99) as compared to those with private insurance. The adjusted r^2^ value for this regression model was 0.17, meaning the variables included in the model accounted for 17% of the variability in telehealth use.

### 3.5. Patient Portal Activation

Finally, we identified demographic and socioeconomic predictors of patient portal activation (Table 6). In unadjusted analysis, Black (OR 0.31, 95% CI: 0.27–0.35), Other (OR 0.26, 95% CI: 0.24–0.29), and Unknown (OR 0.67, 95% CI 0.55–0.81) as compared to White race, and Hispanic/Latine ethnicity (OR 0.33, 95% CI: 0.33–0.35) as compared to non-Hispanic/Latine ethnicity were associated with lower odds of patient portal activation. In adjusted analysis, Black (aOR 0.57, 95% CI: 0.49–0.66), Other (aOR 0.70, 95% CI: 0.62–0.79), and Unknown (aOR 0.73, 95% CI: 0.63–0.79) races remained statistically significant compared to White race. However, upon inclusion of an interaction term between race and income, only Other race (aOR 0.58, 95% CI: 0.45–0.75) was found to be statistically significantly associated with lower odds of patient portal activation. Additionally, Hispanic/Latine ethnicity was no longer statistically significant (aOR 0.97, 95% CI: 0.85–1.09). In unadjusted and adjusted modeling, public (both CCS and non-CCS) insurance (aOR 0.44, 95% CI: 0.39–0.49; aOR 0.33, 95% CI: 0.30–0.38, respectively) was associated with lower odds of patient portal activation, as compared to those with private insurance. Additionally, the odds of patient portal activation were lowest among those living in the lowest income quartile (aOR 0.68, 95%: 0.53–0.87), as compared to those living in the highest income quartile. Finally, Spanish as primary language (aOR 0.20, 95% CI: 0.17–0.23) and another primary language (aOR 0.34, 95% CI: 0.26–0.45) were associated with lower odds of patient portal activation as compared to English as primary language, in unadjusted and adjusted analysis. The r^2^ value for this regression model was 0.27, meaning the variables included in the model accounted for 27% of the variability in telehealth use.

## 4. Discussion

This study improves our understanding of the demographic, socioeconomic, and clinical characteristics and healthcare utilization patterns of a cohort of youth living with chronic illness seen at a large quaternary care academic medical center in 2021. These youth were found to be high healthcare users, with multiple subspecialty clinic visits, numerous referrals, and high rates of emergency department use and hospitalization. They were prescribed multiple medications but received few supportive or rehabilitative services.

This study provides new insights about the burden of clinic visits. Missed clinic visits have been associated with medication non-adherence [17], which has been associated with higher healthcare utilization among children with chronic illness [18] and worse health outcomes overall [19]. Distance from care, insurance status, and race and ethnicity have been previously identified as predictors of missed clinic appointments, in general, in pediatric practice [20]. Within pediatric subspecialty practice, younger age (less than 12 years), public insurance, and Black race were associated with more missed clinic visits [21,22]. As we hypothesized, our cohort experienced a high number of clinic visits (a median of nine clinic visits per year). This may be an indication of the complexity of care required and likely contributes to the burden of care experienced by both young people and their families. This burden is further compounded by the high number of referrals (median of four), which generate additional medical encounters and engagement with additional providers. Our data show similar trends to prior general and subspecialty pediatric cohorts, including minoritized race (including Black, Native Hawaiian or other Pacific Islander, and Unknown) and public insurance as significant predictors of missed clinic visits, in adjusted analysis.

The racial disparities in missed clinic visits identified are important to highlight. Our adjusted models controlled for insurance status and other measures of socioeconomic status, suggesting other unmeasured variables associated with race driving these associations. We would posit that these unmeasured variables are likely associated with or influenced by forces of structural racism that may hinder access to care. For example, in the United States, residential segregation is pervasive, with most individuals living in areas that are economically and racially divided [23]. This, in turn, may lead to unequal access to high-quality education and employment. For such individuals, missed work for medical appointments (whether in person or by telehealth) may result in significant financial strain. This may be one mechanism by which Black and other minoritized youth experience higher missed clinic visits; these complexities may not be fully accounted for in our statistical models. Qualitative interviews with these youth and their caregivers could help improve our understanding of individual-, community-, and societal-level barriers to care that, in turn, could inform effective interventions. Additionally, further exploration of other measures that quantify racial segregation in the U.S., such as redlining scores [24], would improve our understanding of forces of structural racism that may underlie some of the findings of our study.

Predictors of telehealth use were also of interest in this study, as telehealth-based care coordination has been previously demonstrated to significantly decrease healthcare utilization within a complex pediatric population [25]. Additionally, telehealth initiatives have been shown in pediatric cohorts to ameliorate missed appointments [26]. In our study, we did not identify racial or socioeconomic health disparities impacting telehealth use, though racial disparities affecting missed clinic visits were identified. Our findings suggest that telehealth use is not currently sufficient to attenuate the racial disparities impacting missed clinic visits. This contrasts with prior studies that found telehealth use was associated with fewer missed appointments, including those with chronic illnesses [27]. Therefore, potential interventions aimed at improving access to telehealth, such as providing hotspots or other means for internet access, may not be sufficient to ameliorate these disparities. While telehealth is an important tool for medication adherence, improved disease control, and, in turn, lower emergency department use and hospitalizations [28,29,30], more work will be needed to understand the root causes of such disparities in missed clinic visits, such as missed work and compensation for a guardian or missed school or other commitments for youth. We were not able to assess if there were differences in telehealth offered by race or ethnicity. It is possible that implicit biases of individual providers or other staff who schedule appointments or other structural forces could influence whether a telehealth visit is offered and may be a potential mechanism of these racial disparities. This was not addressed by our study and should be the subject of future work.

We identified lower patient portal activation for those with public insurance, lower income, and primary Spanish speakers. Patient portal use has been shown to improve patient outcomes and improve patient engagement in preventative health measures [31], which are critically important for youth with chronic illness. Nevertheless, the literature demonstrates striking racial, language, and socioeconomic health disparities [32], also highlighted in our study. Importantly, prior work has also demonstrated public interest in patient portal use, even among those who do not speak English [33,34]. One might expect that the rates of patient portal activation would be high for frequent users of the health system, yet only two-thirds of the individuals in our cohort had an active account. Given the importance of patient portals, especially for high users of the health system, clinic-level and healthcare system-level efforts to improve patient portal access, such as automated enrollment programs [35], should be a key priority for youth with chronic illnesses who interact with and use the healthcare system frequently, and may limit the socioeconomic and language disparities in patient portal activation experienced by our cohort.

Youth in this cohort had a wide range of diseases and were receiving care from multiple pediatric subspecialty clinics. While our cohort included common chronic diagnoses of childhood such as type I diabetes mellitus and mental health disorders, other common pediatric diseases like asthma and eczema were less represented. Of note, gender dysphoria was the fifth most common diagnosis. This highlights that gender dysphoria may be an underrecognized chronic condition affecting youth who are high users of the healthcare system. Prior studies have suggested a prevalence of about 0.5–1.3% for gender dysphoria among all children [36] whose care necessitates frequent interaction with the healthcare system. Our findings may reflect the availability of specialized care for young transgender youth through a dedicated subspecialty clinic at our center. Such clinics have been associated with significant improvements in transgender youth mental health [37].

Other key findings included a high burden of prescribed medications in our cohort. Those young people with chronic conditions were prescribed a median of six medications, a striking number for a population in which polypharmacy has been defined as two or more medications [38]. Medication-related problems, including drug–drug interactions and adverse effects, were common in another pediatric cohort of patients on at least five or more medications [39]. The implications of the daily and potentially lifelong medication burden must be considered and addressed if treatment is to be effective and sustained. Partnerships with pharmacists, nurses, and others to anticipate and address drug interactions, prevent and manage symptoms and side effects, and address developmental and lifestyle issues that might interfere with consistent and effective medication use are important considerations for the management of pediatric chronic illnesses [39].

A potential limitation of our study is generalizability to individuals with lower healthcare utilization and those receiving care at other centers. Most of our cohort lived in the San Francisco Bay Area (72%), which may limit generalizability to youth coming from other areas of the United States or other countries. Although we anticipated a majority local cohort, we were surprised to find less than expected diversity within our population from a community demographics perspective. For example, our cohort of patients mainly came from highly affluent areas with a high median income for zip code across the cohort at large of over USD 100,000 per year, much higher than the average median income in the United States of USD 70,784 and USD 91,905 in California for the year 2021 [16]. Using a geocoded measure of social and economic deprivation linked to a 5-digit zip code called the Multidimensional Deprivation Index (MDI) [13], our cohort lived in highly advantaged areas with scores approximating the best score of 0. Additionally, our cohort lived in highly urban areas as defined by RUCA scores from the USDA [12]. Importantly, each of these measures was derived from relatively large geographic areas linked closely to 5-digit zip codes and may therefore misclassify smaller areas of social deprivation connected to indices linked to census tracts or blocks, including the area deprivation index (ADI) [40,41]. However, our medical center serves a large catchment area for a broad geographic region at two children’s hospitals and surrounding outreach clinics across the region. Limitations also include a high likelihood of unmeasured variables influencing our outcomes, as suggested by low r^2^ values in our multivariable models, most notably for missed clinic visits. Finally, we may have either missed youth who have a chronic illness but did not have sufficient encounters within our healthcare system or may have misclassified those with a high number of encounters without a significant chronic illness burden. We attempted to account for this by limiting our inclusion criteria to exclude those not likely to reflect a clinical encounter with a provider. The strengths of our study include a large sample size facilitating regression modeling that included multiple covariates and analysis of both individual- and community-level social determinants of health, thereby providing a comprehensive assessment of potential health disparities among high users of pediatric subspecialty care.

Our ongoing work includes an analysis focused on more discerning geocoded measures of social deprivation (such as ADI) to identify patients in the highest need of support services within our institution to guide future programming with a lens of health equity. Qualitative studies are needed to understand the unique challenges and disparities along factors that may contribute to gaps in care that disproportionately affect those of a minoritized race and are experienced by youth and their families who frequently interact with large academic pediatric medical systems. Similar analyses performed at other large academic centers would also be helpful in ascertaining if there are geographic trends impacting the types of patients seen and their healthcare utilization patterns.

## 5. Conclusions

In our study of youth with chronic illness, we identified high healthcare utilization, including a high number of clinic visits per year, multiple subspecialty referrals, and many medications. Importantly, we identified striking racial and socioeconomic health disparities among these youth. These included missed clinic visits, which represent important gaps in care, that were not always ameliorated by health technology tools, such as telehealth and patient portals. This growing population in pediatric medicine will require ongoing attention and study. Systematic efforts to maximize healthcare and limit the burden will require a multidisciplinary approach. Our specific next steps in understanding the identified disparities include engagement with this population via qualitative research methods to identify barriers to successfully completing clinic visits and the use of telehealth and the patient portal. We will work with these youth to identify and test novel interventions to improve healthcare delivery that is equitable and limits the burden for this population who interact frequently with the medical system.

## Figures and Tables

**Table 1:** Demographic characteristics of youth (up to age 25) defined as high users of the medical system seen at University of California, San Francisco (UCSF) Health in 2021.

	Total
	N = 14,245
Age (IQR)	14 (8–17)
Sex assigned at birth	
Female	7080 (50%)
Male	7152 (50%)
Non-binary or Unknown	13 (0%)
Race	
White	5371 (38%)
Asian	1689 (12%)
Black	1375 (10%)
American Indian or Alaska Native	150 (1%)
Native Hawaiian or other Pacific Islander	97 (1%)
Other	4998 (35%)
Unknown or Declined to State	565 (3%)
Ethnicity	
Hispanic or Latine	4739 (33%)
Not Hispanic or Latine	9013 (63%)
Unknown or Declined to State	493 (4%)
Primary Language	
English	11,961 (84%)
Spanish	1953 (14%)
Other	331 (2%)
Payor	
Private	6716 (47%)
Public (CCS)	3833 (27%)
Public (Non-CCS)	3573 (25%)
Unknown	123 (1%)

**Table 2:** Community-level characteristics of youth (up to age 25) defined as high users of the medical system seen at University of California, San Francisco (UCSF) Health in 2021.

	Total
	N = 14,245
Median Income for Zip Code (IQR)	USD 108,680 (USD 74,718–142,785)
Median Income Quartiles	
1 (Lowest)	3699 (26%)
2	3464 (24%)
3	3708 (26%)
4 (Highest)	3374 (24%)
Median Multidimensional Deprivation Index (IQR)	0.11 (0.10–0.12)
MDI Quartiles	
1 (Lowest)	4380 (31%)
2	3555 (25%)
3	2778 (20%)
4 (Highest)	3528 (25%)
Missing	4 (0%)
Median Rural–Urban Commuting Area Score (IQR)	1 (1–1)
Total Living in an Urban Population (%)	13,181 (93%)
Live in the Bay Area	10,212 (72%)

**Table 3:** Healthcare utilization patterns of youth (up to age 25) defined as high users of the medical system seen at University of California, San Francisco (UCSF) Health in 2021.

	Total
	N = 14,245
Median number of visits (IQR)	12 (6–25)
Median number of missed visits (IQR)	0 (0–2)
Median number of telehealth visits (IQR)	2 (1–4)
Proportion (%) of total visits as telehealth (IQR)	25 (4–50)
Median number of medications (IQR)	6 (3–10)
Median number of referrals (IQR)	4 (1–8)
Active online portal users (%)	9823 (69%)
Any ED visit (%)	2602 (18%)
Any elective or non-elective hospitalization (%)	2199 (15%)
Any child-life encounter (%)	277 (2%)
Any nutrition encounter (%)	1192 (8%)
Any social work encounter (%)	650 (5%)
Any telehealth use (%)	11,039 (78%)

**Table 4:** Association between key demographic and socioeconomic variables and missed clinic visits (via unadjusted and adjusted Poisson regression modeling) among youth (up to age 25) defined as high users of the medical system seen at University of California, San Francisco (UCSF) Health in 2021.

Demographic and Socioeconomic Predictors	Unadjusted IRR **	95% CI	*p*-Value	Adjusted * IRR **	95% CI	*p*-Value
Race						
White						
Asian	0.83	0.76, 0.91	<0.001	0.83	0.76, 0.91	<0.001
Black	1.90	1.74, 2.08	<0.001	1.56	1.42, 1.71	<0.001
American Indian/Alaska Native	1.30	1.01, 1.66	0.04	1.17	0.94, 1.46	0.15
Native Hawaiian or other Pacific Islander	1.33	1.02, 1.74	0.03	1.28	1.01, 1.62	0.04
Other	1.28	1.20, 1.37	<0.001	1.09	1.01, 1.18	0.02
Unknown/Declined to State	1.19	1.03, 1.38	0.02	1.08	0.90, 1.29	0.42
Ethnicity						
Non-Hispanic/Latine						
Hispanic or Latine	1.09	1.03, 1.16	0.002	1.05	0.98, 1.14	0.16
Unknown/Declined to State	1.01	0.87, 1.17	0.90	1.07	0.88, 1.29	0.51
Primary Payor						
Private						
Public (CCS)	1.55	1.45, 1.65	<0.001	1.54	1.42, 1.68	<0.001
Public (non-CCS)	1.77	1.66, 1.89	<0.001	1.52	1.40, 1.65	<0.001
Unknown	1.07	0.82, 1.39	0.64	1.02	0.78, 1.33	0.87
Median Income Quartile						
1 (Lowest)	1.57	1.46, 1.70	<0.001	1.33	1.21, 1.46	<0.001
2	1.41	1.30, 1.52	<0.001	1.23	1.12, 1.36	<0.001
3	1.37	1.26, 1.48	<0.001	1.20	1.11, 1.31	<0.001
4 (Highest)						
Language						
English						
Spanish	1.03	0.95, 1.11	0.49	0.85	0.78, 0.93	0.001
Other	0.97	0.84, 1.12	0.70	0.92	0.78, 1.08	0.30

* Adjusting for age, sex assigned at birth, any inpatient hospitalization (as a surrogate for disease severity), most frequently visited clinical site, local (defined as living in one of the nine counties comprising the Bay Area), rurality/urbanicity (by RUCA scoring), most frequently visited subspecialty clinic/group. ** Incidence rate ratio.

**Table 5:** Association between key demographic and socioeconomic variables and the number of telehealth clinic visits (via unadjusted and adjusted Poisson regression modeling) among youth (up to age 25) defined as high users of the medical system seen at University of California, San Francisco (UCSF) Health in 2021.

Demographic and Socioeconomic Predictors	Unadjusted IRR **	95% CI	*p*-Value	Adjusted * IRR **	95% CI	*p*-Value
Race						
White						
Asian	0.89	0.82, 0.95	0.001	1.06	0.96, 1.17	0.26
Black	0.63	0.57, 0.69	<0.001	0.87	0.69, 1.09	0.21
American Indian/Alaska Native	0.88	0.65, 1.17	0.38	1.02	0.67, 1.55	0.92
Native Hawaiian or other Pacific Islander	0.73	0.56, 0.95	0.02	0.83	0.48, 1.43	0.50
Other	0.73	0.69, 0.77	<0.001	1.09	0.97, 1.22	0.13
Unknown/Declined to State	1.34	1.21, 1.48	<0.001	1.36	1.16, 1.59	<0.001
Ethnicity						
Non-Hispanic/Latine						
Hispanic or Latine	0.80	0.75, 0.84	<0.001	0.96	0.90, 1.02	0.17
Unknown/Declined to State	1.49	1.32, 1.68	<0.001	0.98	0.85, 1.14	0.80
Primary Payor						
Private						
Public (CCS)	0.62	0.59, 0.66	<0.001	0.90	0.83, 0.99	0.02
Public (non-CCS)	0.82	0.77, 0.88	<0.001	0.96	0.86, 1.07	0.44
Unknown	0.70	0.48, 1.03	0.07	0.94	0.71, 1.26	0.70
Median Income Quartile						
1 (Lowest)	0.73	0.67, 0.78	<0.001	0.91	0.81, 1.02	0.12
2	0.87	0.82, 0.93	<0.001	1.00	0.91, 1.09	0.92
3	0.92	0.85, 0.99	0.02	1.04	0.97, 1.12	0.26
4 (Highest)						
Language						
English						
Spanish	0.76	0.69, 0.83	<0.001	1.02	0.93, 1.11	0.73
Other	0.78	0.68, 0.91	0.001	1.11	0.98, 1.26	0.11

* Adjusting for age, sex assigned at birth, any inpatient hospitalization (as a surrogate for disease severity), most frequently visited clinical site, local (defined as living in one of the nine counties comprising the Bay Area), rurality/urbanicity (by RUCA scoring), most frequently visited subspecialty clinic/group, and including an interaction term between race and median income quartile (*p* = 0.007) and between race and payor (*p* = 0.04). ** Incidence rate ratio.

**Table 6:** Association between key demographic and socioeconomic variables and patient portal (MyChart) activation (via unadjusted and adjusted logistic regression modeling) among youth (up to age 25) defined as high users of the medical system seen at University of California, San Francisco (UCSF) Health in 2021.

Demographic and Socioeconomic Predictors	Unadjusted OR **	95% CI	*p*-Value	Adjusted * OR **	95% CI	*p*-Value
Race						
White						
Asian	1.15	1.00, 1.33	0.05	1.25	0.89, 1.73	0.20
Black	0.31	0.27, 0.35	<0.001	0.90	0.55, 1.46	0.66
American Indian/Alaska Native	1.28	0.82, 2.01	0.28	2.22	0.26, 18.85	0.47
Native Hawaiian or other Pacific Islander	0.75	0.47, 1.20	0.23	0.77	0.16, 3.77	0.75
Other	0.26	0.24, 0.29	<0.001	0.58	0.44, 0.75	<0.001
Unknown/Declined to State	0.67	0.55, 0.81	<0.001	0.67	0.40, 1.09	0.11
Ethnicity						
Non-Hispanic/Latine						
Hispanic or Latine	0.33	0.30, 0.35	<0.001	0.97	0.85, 1.09	0.52
Unknown/Declined to State	1.12	0.90, 1.41	0.30	1.00	0.73, 1.37	0.99
Primary Payor						
Private						
Public (CCS)	0.19	0.17, 0.20	<0.001	0.44	0.39, 0.49	<0.001
Public (non-CCS)	0.15	0.14, 0.17	<0.001	0.33	0.29, 0.38	<0.001
Unknown	0.30	0.21, 0.44	<0.001	0.42	0.28, 0.65	<0.001
Median Income Quartile						
1 (Lowest)	0.19	0.17, 0.22	<0.001	0.68	0.53, 0.88	0.003
2	0.30	0.27, 0.34	<0.001	0.67	0.53, 0.85	0.001
3	0.41	0.36, 0.46	<0.001	0.73	0.59, 0.90	0.003
4 (Highest)						
Language						
English						
Spanish	0.11	0.10, 0.12	<0.001	0.20	0.17, 0.23	<0.001
Other	0.23	0.19, 0.29	<0.001	0.36	0.25, 0.52	<0.001

* Adjusting for age, sex assigned at birth, any inpatient hospitalization (as a surrogate for disease severity), most frequently visited clinical site, local (defined as living in one of the nine counties comprising the Bay Area), rurality/urbanicity (by RUCA scoring), most frequently visited subspecialty clinic/group, and including an interaction term between race and median income quartile (*p* = 0.006). ** Odds ratio.

## Data Availability

The raw data supporting the conclusions of this article will be made available by the authors upon request.
